# Subtotal thymectomy concomitant with emergency coronary revascularization in a myasthenia gravis case with severe left main stem stenosis

**DOI:** 10.1186/1749-8090-8-S1-P182

**Published:** 2013-09-11

**Authors:** U Yetkin, H Cakir, N Karakas, M Aksun, EB Ozturk, Y Beckmann, A Calli, I Yurekli, V Kuru, A Gurbuz

**Affiliations:** 1 Department of Cardiovascular Surgery, Izmir Katip Celebi University Ataturk Training and Research Hospital, Izmir, Turkey; 2 Department of Anesthesiology, Izmir Katip Celebi University Ataturk Training and Research Hospital, Izmir, Turkey; 3 Department of Neurology, Izmir Katip Celebi University Ataturk Training and Research Hospital, Izmir, Turkey; 4 Department of Clinical Pathology, Izmir Katip Celebi University Ataturk Training and Research Hospital, Izmir, Turkey

## Background

Thymectomy is an exclusive treatment modality for myasthenia gravis (MG).

## Methods

Our case was a 69-year-old male. He was diagnosed as myasthenia gravis a year ago and oral pyridostigmine plus corticosteroid therapy was initiated. Three months ago, he received intravenous immunoglobulin (IVIG) therapy for 5 days. Monthly repeated doses of IVIG were planned but he developed cardiac syncope. He underwent coronary angiography revealing multiple significant coronary stenoses at another institution. Transthoracic echocardiography showed an ejection fraction of 40% as consistent with ventriculography. Left ventricle was hypertrophied (left ventricular end-diastolic and en-systolic diameters were 61 and 49 mm) and interventricular septal thickness was measured as 13 mm. He was presented at our Common Council of Cardiology and Cardiovascular Surgery due to the probability of this condition to cause a contraindication for 5-day IVIG therapy. The decision was a high-risk coronary bypass surgery.

## Results

He was taken into operating room for emergency bypass surgery. The thymus gland remnants were subtotally excised with surrounding pleural fat tissue. Then a standard aortounicaval cannulation was made. Right great sapheonous vein was prepared as a conduit and the distal anastomoses were made at left anterior descending (LAD) artery, second obtuse marginal branch of the circumflex artery and right coronary artery.The histopathological examination of the extirpated tissues concluded that ectopic thymus islets were observed within mediastinal fat tissue.

## Conclusions

Consequently, emergency coronary surgery is becoming more widely used in cases with left main stem disease. Combination of thymectomy with coronary arterial surgery is not usual particularly if an emergency surgery is planned. Cooperation of the neurologist, anesthesiologist and surgeon perioperatively is the key factor in successful treatment at this type of combined interventions.

